# Hepatoprotective activity of petroleum ether, diethyl ether, and methanol extract of *Scoparia dulcis* L. against CCl4-induced acute liver injury in mice

**DOI:** 10.4103/0253-7613.55206

**Published:** 2009-06

**Authors:** T.K. Praveen, S. Dharmaraj, Jitendra Bajaj, S.P. Dhanabal, S. Manimaran, M.J. Nanjan, Rema Razdan

**Affiliations:** TIFAC CORE HD, J.S.S College of Pharmacy, Rocklands, Ooty-643 001, Tamilnadu, India; 1 Department of Pharmacology, Al Ameen College of Pharmacy, Bangalore-560027, Karnataka, India

**Keywords:** Carbon tetrachloride, hepatoprotective activity, *Scoparia dulcis* L.

## Abstract

**Objectives:**

The present study was aimed at assessing the hepatoprotective activity of 1:1:1 petroleum ether, diethyl ether, and methanol (PDM) extract of *Scoparia dulcis* L. against carbon tetrachloride-induced acute liver injury in mice.

**Materials and Methods:**

The PDM extract (50, 200, and 800 mg/kg, p.o.) and standard, silymarin (100 mg/kg, p.o) were tested for their antihepatotoxic activity against CCl4-induced acute liver injury in mice. The hepatoprotective activity was evaluated by measuring aspartate aminotransferase, alanine aminotransferase, alkaline phosphatase, and total proteins in serum, glycogen, lipid peroxides, superoxide dismutase, and glutathione reductase levels in liver homogenate and by histopathological analysis of the liver tissue. In addition, the extract was also evaluated for its *in vitro* antioxidant activity using 1, 1-Diphenyl-2-picrylhydrazyl scavenging assay.

**Results:**

The extract at the dose of 800 mg/kg, p.o., significantly prevented CCl4-induced changes in the serum and liver biochemistry (*P* < 0.05) and changes in liver histopathology. The above results are comparable to standard, silymarin (100 mg/kg, p.o.). In the *in vitro* 1, 1-diphenyl-2-picrylhydrazyl scavenging assay, the extract showed good free radical scavenging potential (IC 50 38.9 ± 1.0 μg/ml).

**Conclusions:**

The results of the study indicate that the PDM extract of *Scoparia dulcis* L. possesses potential hepatoprotective activity, which may be attributed to its free radical scavenging potential, due to the terpenoid constituents.

## Introduction

Herbal drugs play a major role in the treatment of hepatic disorders. A number of medicinal plants and their formulations are widely used for the treatment of these disorders.[1,2] In addition to the already existing medicinal plants, there are several unexplored medicinal plants that need to be studied for their therapeutic potential against liver disorders. *Scoparia dulcis* linn (family: Scrophulariaceae) is a glabrous under shrub with small white flowers, commonly found on wastelands and fallow fields. This plant is widely used in the indigenous system of medicine for treating liver ailments.[3,4]

Phytochemical screening of the plant revealed the presence of three different types of diterpenoids; labdane-type, scopadulcic acids A B C, and scopadiol; scopadulan-type, scopadulcic acids A and B, and scopadulciol; and aphidicolin-type, scopadulin.[5] It is also reported to contain flavonoid, 7-O-methyl scutellarein, and an antidiabetic compound, amellin.[6] In the present study, the terpenoid extract of the plant was prepared using PDM and evaluated for its hepatoprotective activity against CCl4-induced acute liver injury in mice and *in vitro* free radical scavenging activity.

## Materials and Methods

### Drugs and chemicals

Silymarin was a gift sample from Micro Laboratories, Hosur, India. Aspartate amino transferase (ASAT) and alanine amino transferase (ALAT), alkaline phosphatase (ALP) and total proteins (TP), and superoxide dismutase (SOD) and glutathione reductase (GSHR) kits were from RANDOX Laboratories Ltd., United Kingdom. All other chemicals and reagents used were of analytical grade.

### Preparation of PDM extract

The terpenoid extract of *Scoparia dulcis* L. was prepared using PDM, as reported earlier.[7] The whole authenticated (voucher no. BUB12005) plant was collected from the campus of the Manipal College of Pharmacy, Udupi, India. The whole fresh plant was dried under shade at room temperature for seven days and then reduced to coarse powder (sieve no.10/40). This powder was used for the preparation of the PDM extract by the soxhlet extraction method. The dried powder (125 g) was extracted thrice with 750 ml of PDM at 50-55°C for 24 hours. The extract obtained was concentrated at 50°C for 12 hours to obtain a greenish brown residue (Yield 7.2 % w/w).

### Phytochemical analysis of PDM extract

The PDM extract was analyzed for the presence of phytochemicals by qualitative analysis.[8] It was then subjected to High Performance Thin Layer Chromatography (HPTLC) analysis (CAMAG, Muttenz, Switzerland) using precoated Thin Layer Chromatography (TLC) plates of silica gel 60 F254 (Merck, Darmstadt, Germany). A solution of PDM extract (5 mg/ml) was spotted using the Linomat IV applicator. TLC was developed using petether-diethylether-methanol (5:4:1) as a solvent system. After development, the TLC plate was dried and scanned using the CAMAG HPTLC scanner III, integrated with WIN CATS software (Version 4.06), and the peaks were recorded at a wavelength of 254 nm.

### 1, 1-Diphenyl-2-picrylhydrazyl [DPPH] scavenging assay

The assay was carried out in 96 well microplates. An extract of 100 μl, and the standard was serially diluted with double distilled water. To all the wells (except for blank) 100 μl of DPPH (100 mM) solution was added and incubated at room temperature for 20 minutes. The absorbance was measured at 490 nm using ELISA reader (BIORAD-550).

### In vivo antihepatotoxic activity

Swiss albino mice (25-30 g) were procured from an in-house animal facility of J.S.S. College of Pharmacy, Ootacamund. The animals were housed under standard conditions of temperature (22 ± 3°C) and relative humidity (30-70%) with a 12:12 light: dark cycle. The animals were fed with standard pellet diet (Amrit Feeds Ltd. Bangalore.) and water ad libitum. The Institutional Animal Ethics Committee (IAEC) approved the protocol (Proposal no. JSSCP / IAEC / M. Pharm/phyto/03/2007-08).

The *in vivo* antihepatotoxic activity of the PDM extract was carried out against CCl4-induced hepatotoxicity in Swiss albino mice. Thirty-six male Swiss albino mice (30-35 g) were randomized into six groups of six each. Groups 1 and 2 received 0.5% CMC, at a dose of 10 ml/kg, p.o. and served as the normal and control groups, respectively. Groups 3 to 5 received PDM extract at a dose of 50, 200, and 800 mg/kg, p.o., respectively. Group 6 received standard, silymarin 100 mg/kg, p.o. All the groups received assigned treatments for a period of seven days. On day seven all animals except Group 1, received intraperitoneal injection of 50% CCl4 in liquid paraffin (2 ml/kg). Twenty-four hours after CCl4 injection, all the animals were anesthetized using pentobarbitone (35 mg/kg, i.p.). The abdominal artery was isolated and about 0.5 ml of blood was collected by using a 24 gauge hypodermic needle. The blood was allowed to clot for 30 minutes at room temperature and the serum was separated by centrifugation at 2500 rpm for 15 minutes and used for biochemical estimations.

After collection of the blood, the liver was removed from each mouse and washed several times with normal saline. Part of the liver was taken for biochemical estimations and the remaining tissue was preserved in 10% v/v formal saline buffer for histopathological analysis.

### Biochemical estimation

The activities of serum aspartate aminotransferase (ASAT), alanine aminotransferase (ALAT), alkaline phosphatase (ALP), and total proteins (TP) were analyzed using commercial kits, to assess the acute hepatic damage caused by CCl4.

The liver tissue was suspended in 10% w/v ice-cold 0.1M phosphate buffer (pH 7.4), cut into small pieces, and the required quantity was weighed and homogenized using a teflon homogenizer. The homogenate was used for the estimation of enzymic, and non-enzymic antioxidants like superoxide dismutase (SOD), glutathione reductase (GSHR), and lipid peroxide level.

### Histopathological studies

The liver tissue was collected and immediately fixed in 10% formalin, dehydrated in gradual ethanol (50-100%), cleared in xylene and embedded in paraffin. Sections (4-5 μm) were prepared and then stained with hematoxylin and eosin (H and E) dye for photomicroscopic observations.

### Statistical analysis

The data was represented as mean ± S.E.M. Results were analyzed statistically by one-way ANOVA followed by Dunnett's multiple comparison test using Prism software (Version 4). The minimum level of significance was set at *P* < 0.05.

**Track 2, ID:** 

Peak	Start position	Start height	Max position	Max height	Max %	End position	End heigh	Area	Area %	Assigned substance
1	0.00 R1	0.6 AU	0.10 R1	233.9 AU	33.14	0.16 R1	55.6 AU	79583 AU	33.73	Autogenerated
2	0.16 R1	55.8 AU	0.20 R1	116.9 AU	16.56	0.21 R1	10.2 Au	3093.0 AU	13.11	Autogenerated
3	0.21 R1	110.9 AU	0.23 R1	129.0 AU	18.27	0.28 R1	478.9 AU	3954.4 AU	16.76	Autogenerated
4	0.33 R1	49.2 AU	0.37 R1	74.4 AU	10.54	0.42 R1	19.7 AU	3130.1 AU	13.27	Autogenerated
5	0.44 R1	21.2 AU	0.48 R1	27.0 AU	3.82	0.53 R1	8.4 AU	1238.3 AU	5.25	Autogenerated
6	0.53 R1	8.4 AU	0.56 R1	13.6 AU	1.92	0.64 R1	0.5 AU	531.9 AU	2.25	Autogenerated
7	0.65 R1	0.0 AU	0.71 R1	24.2 AU	3.43	0.73 R1	22.6 AU	683.7 AU	2.90	Autogenerated
8	0.73 R1	22.8 AU	0.74 R1	23.8 AU	3.37	0.80 R1	0.0 AU	565.1 AU	2.40	Autogenerated
9	0.80 R1	0.1 AU	0.86 R1	631.AU	8.94	0.93 R1	0.0 AU	2437.2 AU	10.33	Autogenerated

## Results

### Phytochemical analysis of PDM extract

**Figure 1:**
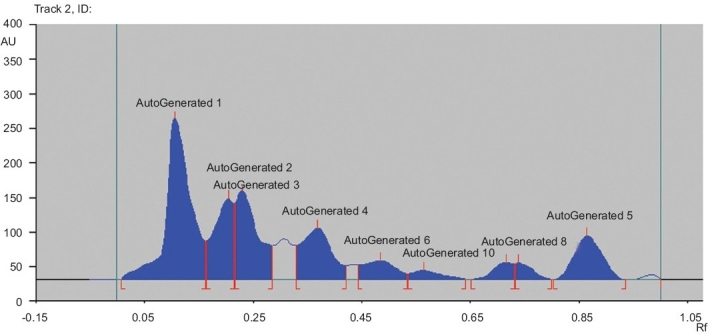
HPTLC finger printing of PDM extract

The qualitative phytochemical analysis of the PDM extract showed the presence of terpenoids and phytosterols. The HPTLC densitometric chromatogram of the PDM extract, at 254 nm, showed nine well-resolved peaks [Figure 1].

### 1, 1-Diphenyl-2-picrylhydrazyl [DPPH] scavenging assay

**Figure 2:**
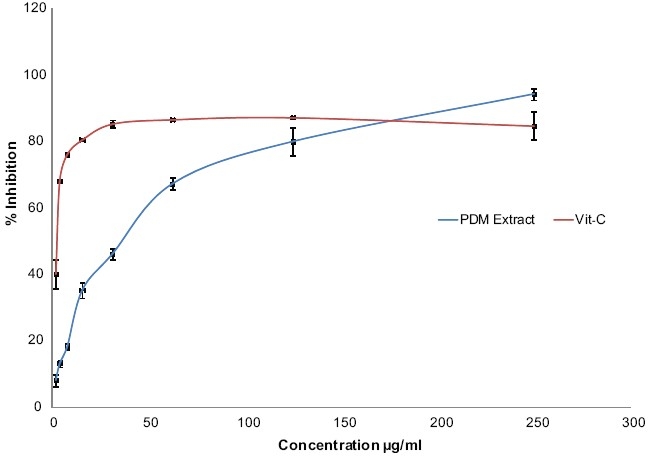
Effect of the PDM extract of *Scoparia dulcis* L. on the DPPH radical scavenging activity

The PDM extract scavenged DPPH radicals in a dose-dependent manner [Figure 2]. The extract at a concentration of 38.9 ± 1.0 μg/ml (mean ± SEM) quenched 50% of the DPPH-free radicals. The standard, vitamin C at a concentration of 2.5 ± 0.5 μg/ml (mean ± SEM) quenched 50% of the DPPH free radicals [Table 1].

**Table 1:** Effect of PDM extract on DPPH radical scavenging activity

	*IC 50 (μg/ml)*
PDM extract	38.9 ± 1.0
Vitamin C	2.5 ± 0.5

Values are mean ± SEM, n = 3

### In vivo antihepatotoxic activity

The PDM extract showed dose-dependent protection against CCl4-induced liver injury. The extract at the highest dose employed, (800 mg/kg, p.o.), significantly prevented carbon tetrachloride-induced elevation in serum ASAT, ALAT, ALP, and TP levels and significantly prevented the decrease in SOD, GSHR, and glycogen, and elevation in lipid peroxide levels in the liver tissue (*P* < 0.05). Standard silymarin at a dose of 100 mg/kg, p.o. also significantly prevented all these changes induced by CCl4 (*P* < 0.05) [Table 2]. The extract at dose levels of 50 mg/kg, p.o and 200 mg/kg, p.o. showed only a moderate degree of protection. At a dose of 50 mg/kg, p.o., the extract showed a significant effect on the ASAT levels. At a dose of 200 mg/kg, p.o., the extract showed significant effects on the ASAT, ALAT, ALP, liver glycogen, and lipid peroxides levels (*P* < 0.05).

**Table 2:** Effect of PDM extract on CCl4-induced acute liver damage

	GP 1:Normal	GP 2:Control	GP 3: PDM 50 mg/kg, p.o.	GP 4: PDM 200 mg/kg, p.o.	GP 5: PDM 800 mg/kg, p.o.	GP 6: Silymarin 100 mg/kg, p.o.
Serum ASAT (u/l)	68.8 ± 2.80	126.8 ± 14.26	85.0 ± 4.50*	75.3 ± 5.51*	73.6 ± 4.16*	81.6 ± 3.51*
Serum ALAT (u/l)	39.7 ± 4.1	93.8 ± 6.4	75.7 ± 7.0	69.7 ± 6.4*	62.5 ± 6.6*	68.3 ± 6.7*
Serum ALP (u/l)	190.1 ± 49.2	427.8 ± 28.8	306.3 ± 12.7	253.8 ± 26.9*	240.6 ± 35.8*	181.6 ± 34.7*
Serum TP (g/dl)	7.1 ± 0.52	4.0 ± 0.38	5.1 ± 0.93	6.1 ± 0.66	6.6 ± 0.44*	7.0 ± 0.32*
Liver GSHR (u/l)	735.8 ± 31.53	366.2 ± 54.51	609.5 ± 54.51	699.2 ± 116.13	752.4 ± 37.8*	795.6 ± 175.8*
Liver SOD (u/ml)	101.3 ± 4.6	44.8 ± 9.3	66.7 ± 6.1	63.0 ± 13.4	89.3 ± 7.5*	81.8 ± 8.0*
Liver glycogen (g/100 of tissue)	0.296 ± 0.01	0.144 ± 0.008	0.153 ± 0.005	0.205 ± 0.006*	0.201 ± 0.009*	0.205 ± 0.004*
Liver MDA (nmol/mg tissue)	0.057 ± 0.006	0.123 ± 0.008	0.101 ± 0.01	0.085 ± 0.010*	0.072 ± 0.005*	0.070 ± 0.008*

Values are mean ± SEM, n = 6

* : *P* < 0.05 when compared to group 2

**Figure 3:**
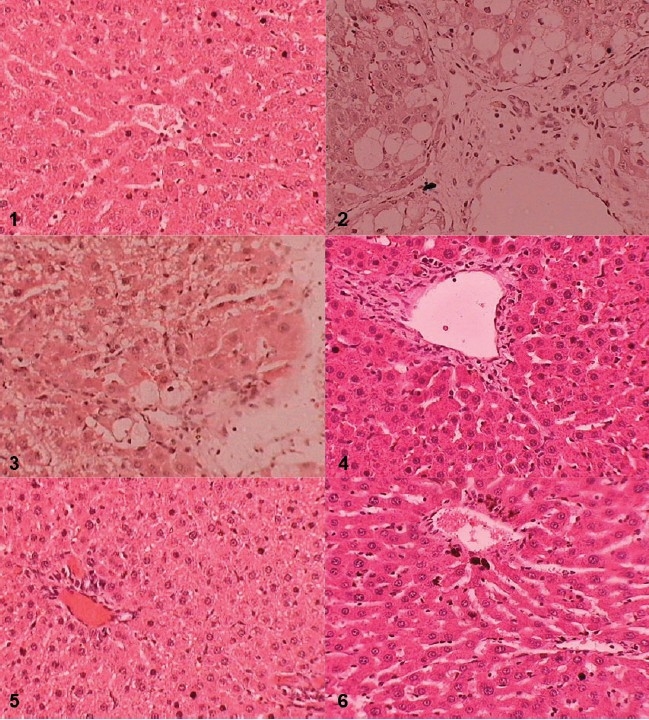
Effect of PDM extract on liver histopathology

The histopathological analysis of the liver tissue also revealed dose-dependent protection against CCl4-induced changes in the liver histology, such as fatty degeneration, lymphatic infiltration, and necrosis. The maximum protection was seen at a dose of 800 mg/kg, p.o. Standard silymarin (100 mg/kg, p.o.) also showed protective activity against CCl4-induced changes in liver histopathology [Figure 3].

## Discussion

In the present study the PDM extract has shown dose-dependent hepatoprotective activity against CCl4-induced acute liver injury. The extract at the highest dose employed (800 mg/kg, p.o.) showed maximum protection against CCl4-induced changes in serum biochemistry, liver antioxidant enzymes, liver peroxide levels, and histopathology. The above activity was comparable to standard, silymarin (100 mg/kg, p.o.) [Figure 3, Table 2]. In addition, the extract also showed appreciable *in vitro* DPPH free radical scavenging activity [Figure 2, Table 1]. These results indicate that the PDM extract possesses both *in vitro* and *in vivo* antioxidant activities, as has been reported by previous studies.[9–12] The hepatotoxic effect of CCl4 has been reported to be due to its metabolite CCl3°, a free radical that alkylates cellular proteins and other macromolecules.[13] The hepatoprotective activity of the PDM extract may be, therefore, attributed to its free radical scavenging potential.

The *Scoparia dulcis* L has been reported to contain three different types of diterpenoids; labdane-type, scopadulcic acids A B C, and scopadiol; scopadulan-type, scopadulcic acids A and B, and scopadulciol; aphidicolin-type, scopadulin.[5] The phytochemical screening of the PDM extract showed the presence of terpenoids. Several compounds belonging to the class of terpenes have been reported to possess antioxidant properties, such as, monoterpenes hydrocarbons (mycene, terpinolene, pinene), oxygenated monoterpenes (nerol, geraniol, linalol, thymol), sesquiterpene hydrocarbons (humulene, valencene, calarene), oxygenated sesquiterpenes (trans-trans-farnesol, farnesol, farnesyl acetate, guaiol), diterpene hydrocarbons (phytol, abetine), and tetraterpene hydrocarbons (caratenoids).[14–16] The free radical scavenging activity of the PDM extract may thus be attributed to its terpenoid content. Further studies to isolate and evaluate these components are in progress.

## Conclusions

The results of the present study indicate that the PDM extract of *Scoparia dulcis* L. possesses potential hepatoprotective activity against CCl4-induced acute liver injury and this may be attributed to its free radical scavenging potential, by the terpenoid content.
